# Supplementary material for: Reconstructing 12-lead ECG from 3-lead ECG using variational autoencoder to improve cardiac disease detection of wearable ECG devices
Source: PLOS Digit Health. 2026 May 22;5(5):e0001335. doi: 10.1371/journal.pdig.0001335 (PMC13196927; doi:10.1371/journal.pdig.0001335)
Supplement: S1 Table — This table presents additional clinical cases used in the physician Turing test, comparing myocardial infarction localization results from multiple cardiologists and the proposed model to further assess model-assisted diagnostic consistency. (PDF) [file pdig.0001335.s001.pdf]

S1 Table. Additional Case Study Samples

In this section, we provide additional case study samples to further illustrate the effectiveness of model-generated ECGs in assisting physicians with myocardial infarction (MI) localization. These samples complement the representative cases presented in the main text and highlight the model's potential to support clinical decision-making by accurately reconstructing infarct regions.

Table 1: Additional case study samples for model-assisted MI localization.

**Physician 1**	**Physician 2**	**Physician 3**	**Model Prediction**
Anterolateral<br>Inferior	Anterolateral	Anterior	Anterolateral
Anterolateral	Anteroseptal	No MI	Anteroseptal
Anteroseptal	Septal	Anteroseptal<br>Inferior	Anteroseptal
Anterolateral	Anteroseptal	Anterior<br>Anteroseptal	Anteroseptal
Anterolateral<br>Inferior	Anteroseptal	No MI	Anteroseptal
Anterolateral	Inferior	Inferior	Inferior
Inferior<br>Lateral	Inferior	Inferior	Inferior
Lateral	Lateral	Lateral	Inferior<br>Lateral
Septal	Septal	Inferior	Septal
