# Supplementary material for: Reconstructing 12-lead ECG from 3-lead ECG using variational autoencoder to improve cardiac disease detection of wearable ECG devices
Source: PLOS Digit Health. 2026 May 22;5(5):e0001335. doi: 10.1371/journal.pdig.0001335 (PMC13196927; doi:10.1371/journal.pdig.0001335)
Supplement: S1 Fig — Representative examples are shown to qualitatively demonstrate the fidelity of reconstructed waveforms across diverse ECG morphologies beyond those presented in the main text. (PDF) [file pdig.0001335.s002.pdf]

S1 Fig. Additional Reconstruction Examples

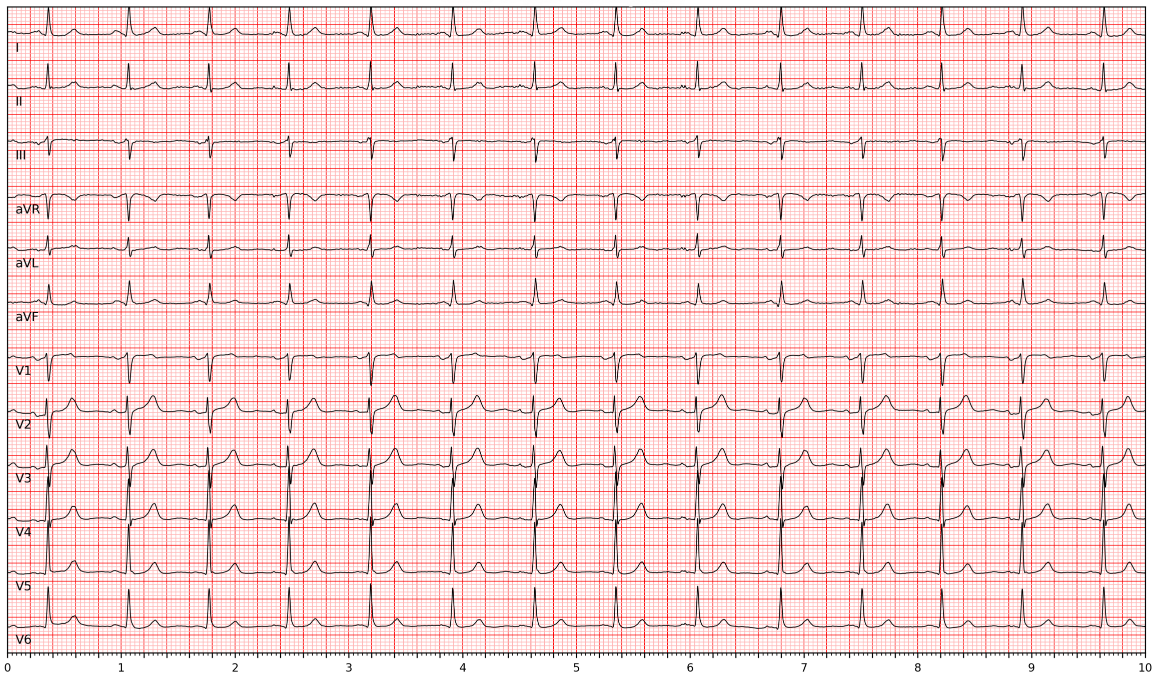

Representative examples of reconstructed 12-lead ECGs from the II/V1/V5 input configuration.

Reconstructions closely match the ground truth morphology, including P-wave, QRS complex, and T-wave patterns. Visual inspection indicates that the reconstructions closely resemble the ground truth across different waveform patterns. These examples illustrate the reconstruction quality of the generative framework under a clinically relevant lead setup.
