# Supplementary material for: Reconstructing 12-lead ECG from 3-lead ECG using variational autoencoder to improve cardiac disease detection of wearable ECG devices
Source: PLOS Digit Health. 2026 May 22;5(5):e0001335. doi: 10.1371/journal.pdig.0001335 (PMC13196927; doi:10.1371/journal.pdig.0001335)
Supplement: S2 Fig — A representative chest-mounted, patch-based electrode layout is illustrated to demonstrate the feasibility of acquiring the proposed reduced-lead configuration in realistic ambulatory settings. (PDF) [file pdig.0001335.s003.pdf]

S2 Fig. Practical Wearable Configuration for II/V1/V5 Acquisition

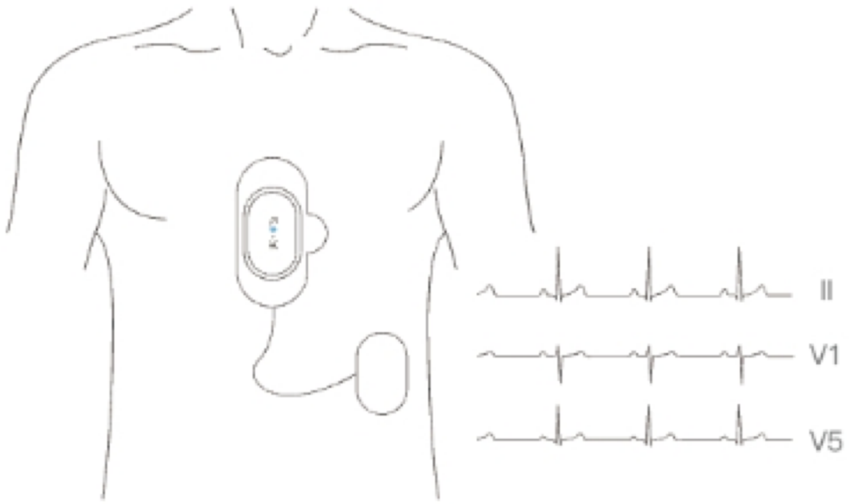

Example of a chest-mounted patch-based electrode layout enabling simultaneous acquisition of modified leads corresponding to II, V1, and V5.

This illustrates a representative wearable ECG configuration that enables simultaneous acquisition of signals corresponding to leads II, V1, and V5 using a chest-mounted patch and auxiliary electrode. This layout adopts a modified Mason–Likar-type arrangement, in which electrodes are placed on the torso to approximate standard limb and precordial leads under ambulatory conditions.

Specifically, the vector formed between the sternal electrode and the inferior-lateral electrode approximates the orientation of Lead II, facilitating reliable rhythm analysis. The sternal electrode provides an effective approximation of the V1 perspective when positioned near the fourth intercostal space, enabling septal and anterior activity monitoring. In addition, the auxiliary electrode located near the left anterior axillary region forms a vector consistent with the standard V5 orientation, capturing lateral left ventricular activity.

Although this configuration employs derived rather than strictly standard 12-lead placements, previous studies and commercial long-term monitoring systems have demonstrated that such torso-based arrangements provide clinically meaningful representations for arrhythmia detection and ischemia monitoring. Minor differences in waveform amplitude and electrical axis may occur due to the absence of true limb electrodes; however, these variations do not substantially affect rhythm interpretation or relative ST-segment assessment.

These practical considerations support the feasibility of deploying the proposed framework on existing patch-based and garment-integrated wearable platforms and demonstrate that the II/V1/V5 input configuration is compatible with realistic ambulatory ECG acquisition scenarios.
