# Supplementary material for: Reconstructing 12-lead ECG from 3-lead ECG using variational autoencoder to improve cardiac disease detection of wearable ECG devices
Source: PLOS Digit Health. 2026 May 22;5(5):e0001335. doi: 10.1371/journal.pdig.0001335 (PMC13196927; doi:10.1371/journal.pdig.0001335)
Supplement: Attachment — Submitted filename: comments.docx [file pdig.0001335.s004.docx]

This manuscript proposes "WearECG," a Variational Autoencoder (VAE) framework designed to reconstruct full 12-lead ECGs from a reduced 3-lead input (specifically leads II, V1, and V5). The authors aim to bridge the gap between portable wearable devices and clinical gold-standard diagnostics. The study utilizes the MIMIC-IV-ECG and PTB-XL datasets for training and validation. The evaluation is comprehensive, utilizing signal-level metrics (MSE, MAE, FID), a downstream diagnostic evaluation using a pre-trained foundation model (ECGFounder), and a Turing test with expert cardiologists.

The paper is well-written, the methodology is sound, and the results demonstrate that the reconstructed signals retain significant diagnostic value, particularly for myocardial infarction localization. However, there are a few minor errors in the text and areas where the discussion could be strengthened before publication.

**Comments:** The authors present a compelling argument for the use of generative models in extending the utility of wearable ECGs. The choice of leads II, V1, and V5 is logically grounded in their orthogonal spatial coverage. The use of a "blinded Turing test" adds valuable clinical perspective to the technical metrics. The manuscript requires only minor revisions to correct typographical errors and clarify specific experimental details.

**Page 11, Line 256 (approximate):** In the paragraph describing the confusion matrix evaluation, the text references "Figure X" instead of the actual figure number. It states: "For the confusion matrix evaluation (Figure X), the model-generated ECG samples...".

**Page 11, Line 242:** There appears to be a sentence fragment or editing error. The text reads: "It also aims to evaluate the feature-level fidelity of the generated electrocardiogram (ECG) signals, we. A balanced set...". Please revise this sentence for grammatical correctness.

In the results section regarding Model-Assisted MI recognition, the text states, "the model-generated ECG samples... were assessed by physicians". Later, it mentions "The model achieved 26 true positives...". It would be beneficial to clarify if the performance metrics (70.9% accuracy) refer to the *physicians'* performance when looking at the generated ECGs, or the *algorithm's* classification performance on those generated ECGs. The phrasing "model-assisted" implies human-in-the-loop, but the metrics seem to describe the classification results. Please clarify this distinction.

**Discussion of Lead Selection:**

The authors select leads II, V1, and V5 because they form an "orthogonal triad". While this is theoretically sound for reconstruction, it would be helpful to briefly discuss the *practicality* of this configuration in current wearable form factors in the Discussion section. Many current consumer wearables (like smartwatches) utilize Lead I or derivations of limb leads. A brief comment on how a V1/V5 configuration might be implemented in a wearable form factor (e.g., patch or chest strap) would strengthen the "wearable" aspect of the title.

**Limitations Section:**

The authors rightly acknowledge that MIMIC data represents a specific patient population. I suggest adding a specific sentence regarding signal noise. Hospital-grade ECGs (even 3-lead subsets) are generally cleaner than ambulatory wearable data, which suffer from motion artifacts. While the authors mention "patient movement" as a challenge , explicitly stating that the *domain gap* between clinical database signals and real-world noisy wearable signals remains a challenge would be a valuable addition to the limitations.

In **Figure 5** (Appendix B), the grid lines on the ECG strips are quite dominant, potentially obscuring the finer details of the P-waves in the reconstruction. If possible, consider lightening the grid lines or increasing the contrast of the signal traces to ensure the morphological fidelity is clearly visible to readers.
