# Supplementary material for: Reconstructing 12-lead ECG from 3-lead ECG using variational autoencoder to improve cardiac disease detection of wearable ECG devices
Source: PLOS Digit Health. 2026 May 22;5(5):e0001335. doi: 10.1371/journal.pdig.0001335 (PMC13196927; doi:10.1371/journal.pdig.0001335)
Supplement: Attachment — Submitted filename: Response to Academic Editor.docx [file pdig.0001335.s005.docx]

**Response to Academic Editor**

Dear Editor,

We sincerely thank you and the editorial team for your careful evaluation of our manuscript and for providing us with the opportunity to revise and resubmit our work.

We are grateful for the constructive comments from the reviewers, which have helped us substantially improve the clarity, rigor, and presentation of the manuscript. We have carefully revised the manuscript to address all concerns raised by the editor and reviewers. In particular, we have clarified evaluation metrics, corrected numerical inconsistencies, strengthened the discussion on lead selection and wearable feasibility, improved textual consistency, and revised the manuscript for accuracy and readability.

We believe that these revisions have significantly strengthened the manuscript, and we sincerely hope that the revised version now meets the high standards of PLOS Digital Health.

Thank you very much for your consideration.

Best regards,
Shenda Hong, on behalf of all authors

**Response to Reviewer #1**

Dear Reviewer,

We sincerely thank you for your positive evaluation of our manuscript and for your valuable and detailed suggestions. We have carefully addressed all your comments and revised the manuscript accordingly. Our point-by-point responses are provided below.

1. FID is typically used for image evaluation. While the authors explain that it is based on features extracted by ECGFounder, they should more elaborately explain why these high-level features were chosen to measure 1D signal distribution similarity and discuss the metric’s interpretability in the ECG domain

**Response:**
We thank the reviewer for this insightful comment. In the revised manuscript, we have expanded the description of FID in Section WearECG can Generate High Fidelity ECG to clarify why high-level features extracted by ECGFounder were adopted. We now explain that ECGFounder is trained under cardiologist supervision and optimized to capture clinically meaningful patterns, such as QRS morphology and ST-segment deviations. Furthermore, we have added a discussion on how FID in this learned feature space provides an interpretable assessment of physiological realism in the ECG domain.

### 2. The authors selected leads II, V1, and V5 and explained their spatial orthogonality. I suggest citing more classic Vectorcardiography theories in the introduction or methods section to strengthen the argument for the optimality of this "three-lead combination" as input.

**Response:**
We thank the reviewer for this valuable suggestion. In the revised manuscript, we have added a discussion of classical vectorcardiography theory and cited foundational works, including the Frank lead system and related models, in Section Method Overview. We now clarify that leads II, V1, and V5 form a quasi-orthogonal triad supported by established vectorcardiographic principles, thereby strengthening the theoretical basis for our lead selection.

3. In the description at line 170, it is stated that "overall MAE and MSE are 0.00100 and 0.01782." However, in Table 1 and the description at line 98, 0.00100 corresponds to MSE, while 0.01782 (or 0.01783) corresponds to MAE. Please rectify this discrepancy.

**Response:**
We thank the reviewer for pointing out this inconsistency. We have carefully reviewed the relevant sections and corrected the values in the main text and Table 1 to ensure consistency. The MAE and MSE values are now accurately reported throughout the manuscript.

4. The titles of Table 4 and Table 5 show inconsistent spelling between "PTBXL" and "PTB-XL." I recommend using "PTB-XL" uniformly throughout the paper.

**Response:**
We appreciate this comment. We have standardized the notation to “PTB-XL” throughout the manuscript, including Tables 4 and 5.

5. The manuscript interchangeably uses "Macro-AUC" (Tables 2, 3) and "Macro-AUROC" (body text). I suggest using Macro-AUROC consistently across all tables and text.

**Response:**
Thank you for this helpful suggestion. We have unified the terminology and now consistently use “Macro-AUROC” in all tables and text.

6. There is an incomplete sentence at line 232 (referenced as line 243): "we . A balanced set..." Please revise this for clarity and grammatical correctness.

**Response:**
We thank the reviewer for identifying this error. The sentence has been revised for grammatical correctness and clarity in the revised manuscript .

7. In the text (around line 242/253), there is a reference to a "confusion matrix (Figure X)," but the corresponding figure is subsequently labeled as Figure 3. Please correct this placeholder in the document.

**Response:**
We appreciate this careful observation. The placeholder has been corrected, and the figure is now properly referenced as Figure 3 in the revised version.

Thank you again for your valuable feedback, which has helped improve the quality of our manuscript.

**Response to Reviewer #2**

Dear Reviewer,

We sincerely thank you for your positive assessment of our work and for your constructive suggestions. We have carefully revised the manuscript to address all the issues raised. Our responses are provided below.

1. The text references “Figure X” instead of the actual figure number.

**Response:**
We appreciate this careful observation. The placeholder has been corrected, and the figure is now properly referenced as Figure 3 in the revised version.

2. There is a sentence fragment:
   “It also aims to evaluate the feature-level fidelity… we. A balanced set…”

**Response:**
We thank the reviewer for identifying this error. The sentence has been revised for grammatical correctness and clarity in the revised manuscript .

3. Please clarify whether the reported performance metrics refer to physician performance or algorithmic classification.

**Response:**
We sincerely thank the reviewer for raising this important point regarding the interpretation of the reported performance metrics.

In the revised manuscript, we have clarified that all confusion matrix results and corresponding performance metrics in this section are based exclusively on physicians’ diagnostic decisions when interpreting the reconstructed ECG signals. During this evaluation, physicians had no access to the original ECG recordings or any algorithmic predictions.

Specifically, the reported accuracy, sensitivity, specificity, precision, and F1-score reflect physician diagnostic performance on the model-generated ECGs, rather than the classification performance of any automated algorithm.

We have revised the corresponding paragraph to explicitly state this distinction and to avoid any potential ambiguity.

4. A brief comment on how a V1/V5 configuration might be implemented in a wearable form factor (e.g., patch or chest strap) would strengthen the "wearable" aspect of the title.

**Response:**
We thank the reviewer for this valuable suggestion. In the revised manuscript, we have added a discussion of classical vectorcardiography theory and cited foundational works, including the Frank lead system and related models, in Section Method Overview. We now clarify that leads II, V1, and V5 form a quasi-orthogonal triad supported by established vectorcardiographic principles, thereby strengthening the theoretical basis for our lead selection.

5. Please explicitly address the domain gap caused by noise and motion artifacts in wearable ECGs.

**Response:**
We sincerely thank the reviewer for this valuable and insightful suggestion.

In the revised manuscript, we have expanded the Limitations section to explicitly address the domain gap between hospital-grade ECG recordings and real-world wearable signals. We now emphasize that clinical database ECGs are generally cleaner than ambulatory recordings, which are often affected by motion artifacts, electrode displacement, and environmental noise.

6. If possible, consider lightening the grid lines or increasing the contrast of the signal traces to ensure the morphological fidelity is clearly visible to readers.

**Response:**
We appreciate this helpful suggestion. We have revised Figure 5 in Appendix B by reducing grid-line intensity and enhancing signal contrast to improve visual clarity.

Thank you again for your valuable feedback.

## **Response to Reviewer #3**

Dear Reviewer,

We sincerely thank you for the thorough and positive summary of our work and for recognizing the methodological rigor, clinical relevance, and comprehensive evaluation framework. We greatly appreciate the constructive comments and have carefully revised the manuscript to address all concerns raised..

1. The feasibility of acquiring leads II, V1, and V5 simultaneously in wearable devices should be discussed.

**Response:**
We sincerely thank the reviewer for this valuable and constructive suggestion.

In the revised manuscript, we have addressed both the theoretical rationale and the practical feasibility of acquiring leads II, V1, and V5 in wearable settings. We now clarify that the simultaneous acquisition of this lead combination has already been demonstrated in existing chest-mounted patch-based and multi-electrode wearable systems.

Specifically, several research-grade and medical-grade wearable ECG platforms have adopted modified electrode layouts (e.g., Mason–Likar–type arrangements) that effectively approximate inferior, septal, and lateral cardiac views corresponding to leads II, V1, and V5 in ambulatory environments. These systems provide practical evidence that the proposed input configuration is technically feasible in real-world deployments.

In addition, we discuss recent advances in flexible electronics, textile-based electrodes, and low-power biosignal acquisition platforms that further facilitate multi-lead sensing in compact wearable form factors. We have also included an appendix illustrating a representative patch-based configuration and its physiological rationale.

These additions strengthen the connection between our proposed method and existing wearable ECG technologies and clarify its applicability in practical clinical and ambulatory settings.

2. Please analyze the high sensitivity and relatively low specificity observed in the confusion matrix.

**Response:**
We thank the reviewer for this insightful comment.

In the revised manuscript, we have provided a more detailed analysis of this performance pattern. We now clarify that the high sensitivity and relatively low specificity may be attributed to multiple factors, including reconstruction uncertainty, physicians’ conservative diagnostic strategies in a screening-oriented setting, and the limited sample size.

We further discuss the potential clinical implications and emphasize the need for future optimization of reconstruction quality and calibration strategies.

3. There is a discrepancy between the reported FID values (12.64 vs. 11.34).

**Response:**
We thank the reviewer for identifying this inconsistency.

After carefully reviewing the manuscript, we found that the discrepancy was caused by an earlier version of the experimental results that was not fully updated in the main text. We have now corrected the FID value throughout the manuscript to ensure consistency with Table 1.

We sincerely appreciate your valuable feedback, which has helped improve our manuscript.
